# Elution and Mechanical Strength of Vancomycin-Loaded Bone Cement: *In Vitro* Study of the Influence of Brand Combination

**DOI:** 10.1371/journal.pone.0166545

**Published:** 2016-11-17

**Authors:** Sheng-Hsun Lee, Ching-Lung Tai, Szu-Yuan Chen, Chih-Hsiang Chang, Yu-Han Chang, Pang-Hsin Hsieh

**Affiliations:** 1 Department of Orthopaedic Surgery, Chang Gung Memorial Hospital, Linkou, No. 5, Fuxing St., Guishan Dist., Taoyuan City, 333, Taiwan, R.O.C; 2 Bone and Joint Research Center, Chang Gung Memorial Hospital, Linkou, No. 5, Fuxing St., Guishan Dist., Taoyuan City, 333, Taiwan, R.O.C; 3 Graduate Institute of Medical Mechatronics, Chang Gung University, No. 259, Wenhua 1st Rd., Guidshan Dist., Taoyuan City, 333, Taiwan, R.O.C; Kyoto Daigaku, JAPAN

## Abstract

Antibiotic-loaded bone cement (ALBC) is widely used in orthopaedic surgery for both prevention and treatment of infection. Little is known about the effect of different brand combinations of antibiotic and bone cement on the elution profile and mechanical strength of ALBC. Standardized specimens that consisted of one of the 4 brands of bone cement and one of the 3 brands of vancomycin were fashioned, producing 12 combinations of ALBC. Two dosages of vancomycin in 40g bone cement were used to represent the high (4g vancomycin) and low (1g vancomycin) dose groups. Concentrations of vancomycin elution from ALBC was measured for up to 336 hours. The ultimate compression strength was tested at axial compression using a material testing machine before and after elution. In both high-dose and low-dose groups, Lyo-Vancin in PALACOS bone cement resulted in the highest cumulative elution and Vanco in Simplex P bone cement resulted in the lowest elution (458% and 65% higher in high- and low-dose groups, respectively). The mechanical strength was not significantly compromised in all groups with low dose vancomycin (range: 70.31 ± 2.74 MPa to 87.28 ± 8.26MPa after elution). However, with the addition of high dose vancomycin, there was a mixed amount of reduction in the ultimate compression strength after cement aging, ranging from 5% (Vanco in Simplex P, 81.10 ± 0.48 MPa after elution) to 38% (Sterile vancomycin in CMW, 60.94 ± 5.74 MPa after elution). We concluded that the selection of brands of vancomycin and bone cement has a great impact on the release efficacy and mechanical strength of ALBC.

## Introduction

Deep infection in orthopaedic surgery is a devastating complication, such as chronic osteomyelitis or periprosthetic joint infection. Surgeries with open procedures always carry the risk of bacterial contamination, which would turn into true infection more easily with the presence of a prosthesis or implant. Thorough debridement, adequate drainage, obliteration of dead space, and long-term systemic antibiotic have been the rule to treat orthopaedic infections. However, toxicity of systemic antibiotic requires serial monitoring of serum antibiotic level. Occasionally, compromised local blood supply reduces antibiotic concentration in the target area [1]. In light of this, local administration of antibiotics is favored in the treatment of orthopaedic infections. Ever since the introduction and increasing popularity of antibiotic-loaded bone cement (ALBC) use in total joint replacement surgery, deep infections have reduced gradually [2]. ALBC has the ability to deliver high concentration of antibiotics over a period of time and is considered an essential part both in the prevention and treatment of periprosthetic joint infection [3]. In addition to antibiotic release, ALBC is also used to fix prosthesis or as a temporary spacer in infected arthroplasty. Hence, the mechanical property of ALBC is also pivotal to a successful orthopaedic surgery.

In order to maximize the effect of ALBC, there were several researches focusing on antibiotic elution capability and mechanical strength of ALBC. Antibiotic elution efficacy has been attributed to several factors, such as type of antibiotic, mixing method, temperature at mixing, combination of different antibiotics, and addition of fillers that would increase porosity of ALBC [3–7]. Mechanical strength of ALBC is dependent on antibiotic dose, type of antibiotic, time of elution, and incorporation of fat or blood [8–10].

To our knowledge, no report has compared the effect of different brands of bone cements and antibiotics on the elution capability and mechanical property of ALBC. Vancomycin added in ALBC is an effective regimen to treat orthopaedic infections[11, 12]. In some circumstances such as revision total joint arthroplasty after eradication of methicillin-resistant Staphylococcal infection, vancomycin-loaded bone cement is also useful for prevention of periprosthetic infection[13, 14]. We attempted to identify if the profiles of vancomycin release and ultimate compression strength are different in 12 combinations of vancomycin-loaded bone cements prepared using 4 different brands of bone cement and 3 different brands of vancomycin.

## Materials and Methods

### Preparation of an antibiotic-loaded cement specimen

We used 4 different types of surgical grade bone cements: Surgical Simplex P (Stryker Orthopedics, Limerick, Ireland), Osteobond (Zimmer, Warsaw, IN), PALACOS R (Heraeus Medical, Newbury, UK), and Depuy-CMW (DePuy CMW, Blackpool, UK); to which vancomycin hydrochloride powders from 3 different manufacturers were added for testing. The 3 vancomycin powders were: Vanco (Gentle Pharmaceutical Co., Yulin, Taiwan), Lyo-Vancin (China Chemical & Pharmaceutical Co., Ltd, Taichung, Taiwan), and Sterile Vancomycin (Hospira Inc., Lake Forest, Illinois, USA).

The vancomycin mixture consisted of high- (4g) or low-dose (1g) vancomycin hydrochloride powder from 4 different manufacturers that was hand-mixed thoroughly with 40 g of powdered bone cement component before the 20 mL liquid monomer component was added. Following the addition of 20 mL of liquid monomer component, the cement-antibiotic mixture was hand-mixed in a ceramic container for 2 minutes to achieve a doughy status and then manually pressed into a plastic mold to form uniform test cylindrical specimens. The cement cylinders, 20 mm in height and 15 mm in diameter, were cured at room temperature for 1 hour.

Twelve groups of vancomycin-loaded bone cement were prepared: Simplex P, Osteobond, PALACOS R, or Depuy CMW bone cement that were loaded with Vanco, Lyo-Vancin, or Sterile Vancomycin. The bone cement with no antibiotic served as a control. Ten specimens were produced in each group.

### Antibiotic elution testing

Six specimens in each group were used to measure antibiotic elution. Each cement cylinder was immersed in a glass tube containing 30 ml of sterile phosphate buffer solution (PBS), kept at 37°C until the designated sampling times, and then the specimen was removed from the test tube. The eluate in each test tube was frozen at −20°C until the analysis for antibiotic concentration. All cement cylinders were washed with 10 mL of PBS and then reimmersed in test tubes containing 30 mL of fresh PBS. Samples were collected at 1, 3, 6, 10, 24, 48, 72, 168, and 336 hours. Antibiotic concentration was determined by fluorescence polarization immunoassay (FPIA) using an Abbot Laboratories TDx Analyzer (Abbot Laboratories, Abbot Park, Ill). FPIA has been used in previous studies with validated results and proved to be more accurate than other assay methods [15, 16]. The lower limit of detection was 1.0 μg/ml for vancomycin.

### Ultimate compression test

Four cement specimens (both before and after 336-hour antibiotic broth elution) in each group were tested for failure in axial compression using the material testing system machine (Bionix 858; MTS Corp., Eden Prairie, MN, US). The specimen was positioned on a flat supporter clamped to the MTS lower wedge grip, and then a stiffened plate was placed on the top surface of specimen. A ball-shape plunger fixed on MTS upper wedge grip was then used to apply compressive force. The experimental setup ensures full surface contact of the specimen to achieve a uniform pressure. Each specimen was compressed at a constant displacement rate of 0.1 mm/s. During testing, parameters involving force, displacement, and time were recorded simultaneously with an increase of 0.05 mm by MTS TestStar software (MTS Corp., MN, USA). The peak force divided by the cross section area (A = π,·r^2^ = 177 mm^2^) of the specimen was defined as “the ultimate compression stress for comparison among the groups.” Bone cement specimens from one of the four brands without antibiotic were used as controls. Four trials in each group were performed, including the control group, and the mean value for the ultimate compression stress of these trials was determined.

### Statistical analysis

The antimicrobial concentration of elution samples and the ultimate compressive stress of cements with different preparations were tested 6 and 4 times, respectively. The results were reported as the mean and standard error. We used an analysis of variance (ANOVA) to determine the statistical difference in the ultimate compression stress and efficiency of antibiotic release between cements with different preparation methods. Tukey’s multiple comparison test was used as a post-hoc test to determine the differences between different groups. A p-value of < 0.05 was considered statistically significant.

## Results

### Antibiotic elution

**Fig 1:**
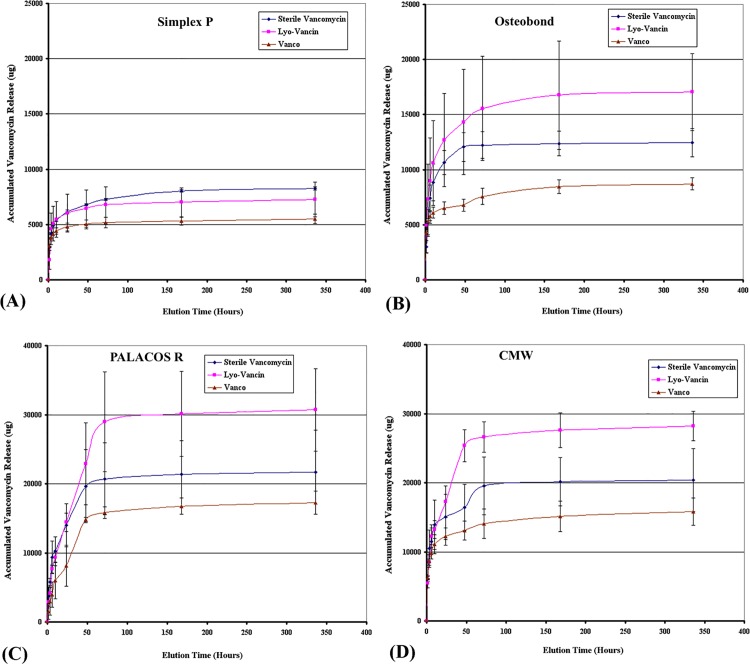
Cumulative antibiotic release amount in high dose group. Graphs showing cumulative antibiotic released from cement specimens of (A) Simplex P; (B) Osteobond; (C) PALACOS R; and (D) CMW loaded with high (4g vancomycin) dose preparations of Sterile Vancomycin, Lyo-Vancin or Vanco in broth elution assay for 336 hours. Values are shown as the mean and standard error of the mean for eight specimens in each group.

**Fig 2:**
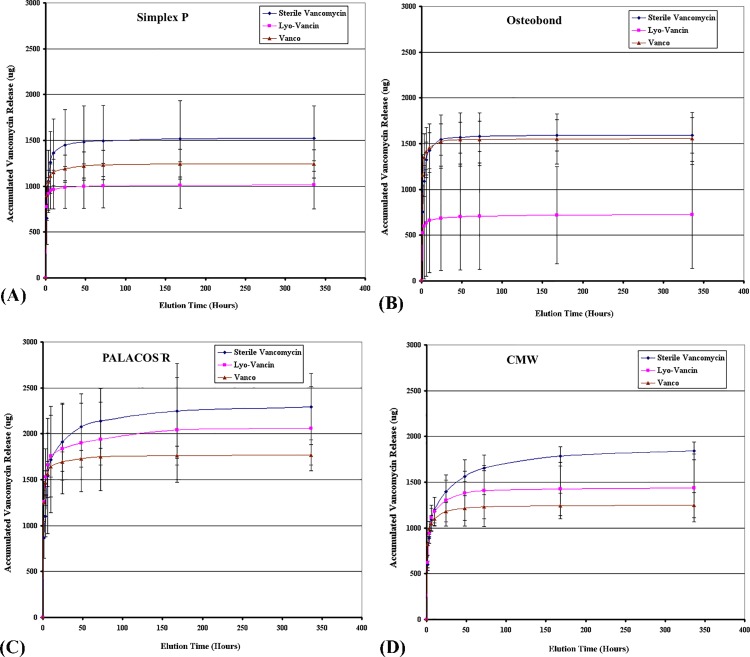
Cumulative antibiotic release amount in low dose group. Graphs showing cumulative antibiotic released from cement specimens of (A) Simplex P; (B) Osteobond; (C) PALACOS R; and (D) CMW loaded with low (1g vancomycin) dose preparations of Sterile Vancomycin, Lyo-Vancin, or Vanco in broth elution assay for 336 hours. Values are shown as the mean and standard error of the mean for eight specimens in each group.

All samples showed burst release during the first time point of the broth elution assay, then reached a plateau after 72 hours (Fig 1 and Fig 2). Elution profiles of cumulative antibiotic released from the different brands of bone cement and different brands of vancomycin with high (4g) and low (1g) dose preparations are shown in Fig 1 and Fig 2, respectively.

#### High dose groups

Osteobond bone cement loaded with Lyo-Vancin had a significantly higher efficiency of antibiotic release than Sterile Vancomycin group and Vanco group (*p* < 0.05). The same results were observed in PALACOS and CMW bone cements, in which Lyo-Vancin added groups showed higher antibiotic elution ability. However, in Simplex P bone cement, the Sterile Vancomycin group had slightly higher release efficacy. When brands of vancomycin were controlled, the release amount was the largest when PALACOS bone cement was used.

#### Low dose groups

Regardless of the brand of bone cement used, antibiotic release was higher when Sterile Vancomycin was added. When the same brand of vancomycin was added, PALACOS bone cement had the largest amount of vancomycin elution.

### Ultimate compression strength

**Fig 3:**
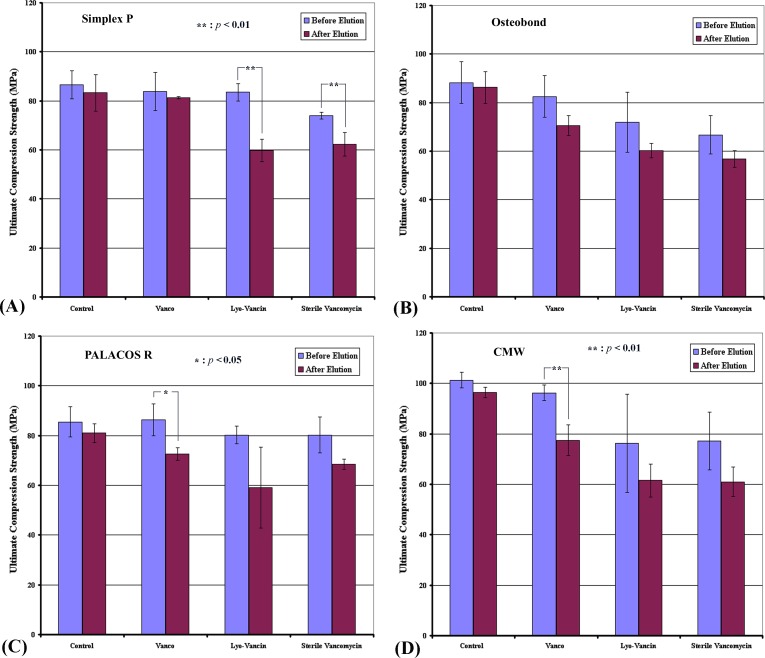
Ultimate compressive strength in high dose group. Mean ultimate compressive strength of (A) Simplex P; (B) Osteobond; (C) PALACOS R; and (D) CMW cement samples loaded with high (4g vancomycin) dose preparations of the 3 brands of antibiotics before and after 336-hour broth elution assay relative to cement without antibiotics (control). Values are shown as the mean and standard error for each group.

**Fig 4:**
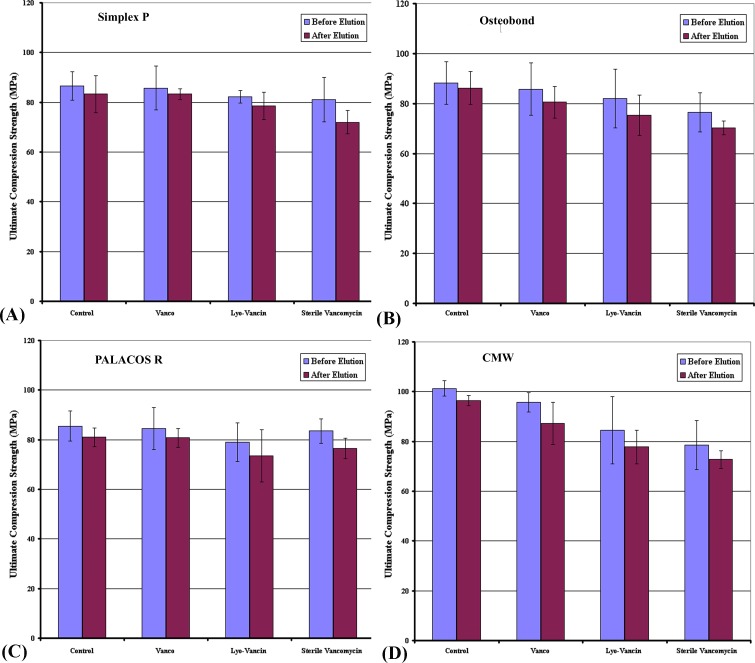
Ultimate compressive strength in low dose group. Mean ultimate compressive strength of (A) Simplex P; (B) Osteobond; (C) PALACOS R; and (D) CMW cement samples loaded with low (1g vancomycin) dose preparations of 3 brands of antibiotics before and after 336-hour broth elution assay relative to cement without antibiotics (control). Values are shown as the mean and standard error for each group.

Mean ultimate compression strength of the 4 brands of bone cements loaded with 3 antibiotics before and after 336-hour broth elution assay relative to the cements without antibiotics are shown in Fig 3 and Fig 4. In the control groups as well as in the other combinations, all brands of bone cement show a significant reduction in ultimate compression strength.

#### High dose groups

The combination with the highest ultimate compression strength after elution was Vanco in Stryker Simplex P bone cement (81.10 ± 0.48 MPa), while that with the lowest was Sterile Vancomycin in Zimmer Osteobond bone cement (56.68 ± 3.74 MPa). Particularly, the mechanical property after elution was better when the antibiotic used was Vanco.

#### Low dose groups

The combination with the highest ultimate compression strength after elution was Vanco in DePuy CMW bone cement (87.28 ± 8.26MPa), while that with the the lowest was sterile Vancomycin in Zimmer Osteobond bone cement (70.31 ± 2.74 MPa). As in the high dose groups, Vanco was a better choice of antibiotic in terms of the mechanical property after elution.

## Discussion

Antibiotic-loaded bone cement (ALBC) has the advantage in the treatment of bone and joint infections because it can deliver high concentrations of local antibiotics, while minimizing systemic toxicity. High dose antibiotic-loaded bone cement (>3.6g per 40g bone cement) is used for beads and spacers to treat existing infection, while low dose ALBC (1 to 2g per 40g bone cement) is used for prosthesis fixation and prophylaxis of infection [17, 18]. *Staphylococcus* species are the most common pathogens in musculoskeletal infections, comprising 50% to 60% [19, 20]. Vancomycin, a glycopeptide, is one of the most commonly used antibiotics in the treatment of methicillin-resistant *Staphylococcus aureus* (MRSA) infection. The use of vancomycin in bone cement, including its stability, elution efficacy, and anti-Staphylococcal ability has been confirmed in previous studies [18].

The dose of antibiotic in ALBC varies depending on the usage. For prophylactic use, antibiotic concentration should not exceed 2g in 40g of polymer. For therapeutic use, many authors recommended 3.6 to 8g in 40g of polymer [17, 21–23]. As a result, in the current study, we used 1g of vancomycin in 40g of bone cement in the low dose groups and 4g of vancomycin in 40g of bone cement in the high dose groups, which represented the prophylactic and therapeutic purpose in clinical use, respectively.

The release of antibiotic from ALBC can range from one to several days [24–26]. It can be influenced by the category and dose of the antibiotic, molecular weight, stability of the antibiotic in the presence of body fluid, bone cement property, mixing method, and curing [7, 27–29]. The antibiotic is released from the surface or cracks and voids of the cement. Due to the hydrophobicity of the polymer, the total amount of antibiotic released is less than 10% of its content, which is released mostly within the first few hours to days [30]. In our study, regardless of the combinations of bone cements and vancomycin, the trends were similar, such that vancomycin elution was high in the first 3 days and reached a plateau thereafter.

There were studies addressing the antibiotic release efficacy based on different brands of bone cement. Many authors found that different brands of bone cement would affect antibiotic elution [24, 25, 31–34], although Marks et al. found no relationship between them.[35] Cerretani et al. studied the efficacy of vancomycin elution in 3 different types of bone cement (CMW1, PALACOS R, and Simplex P). [34] They found that the release is better in the CMW1 group than in the other two. Van de Belt et al. found that the release of antibiotic from Palamed was greater than from PALACOS and CMW. [31] The authors thought that the combination of porosity and roughness of ALBC would influence gentamicin release. Other authors further proved that the initial release is affected by the roughness of the ALBC (the higher the roughness, the greater the release), and the continuous release ability is affected by the porosity of ALBC [16, 22]. Miola et al. conducted an *in vitro* study, which showed that the bone cement powders were similar in size and shape under scanning electron micrograph among the different brands of PMMA and that the compositional analyses did not evidence any significant difference [36]. Commercial bone cements have additional minor contents that serve different purposes, such as benzoyl peroxide as an initiator and zirconium dioxide (ZrO) or barium sulfate as radio-opacifier. ZrO, for example, is also found under scanning electron microscopy in cured bone cements [36]. Whether the amount of ZrO or other minor contents in bone cement would affect the porosity and hence the elution efficacy of ALBC necessitates further investigation.

There are some reports regarding the antimicrobial efficacy of branded and generic vancomycin products when used parentally. Vesga et al. reported inferior therapeutic effect of generic vancomycin despite being the pharmaceutical equivalent to the branded drug [37]. Others found comparative potencies between generic and branded vancomycin, but with some subtle differences [38–40]. They found some impurities existing in generic vancomycin, such as crystalline degradation product-1 (CDP-1) and CDP intermediate. Such byproducts are produced during vancomycin metabolism after deamidation of an asparagine [41]. Accumulation of CDP has been reported to have toxicity or lead to treatment failure [42]. However, to the best of our knowledge, the current literature does not report the effect of different brands of generic vancomycin on the release efficacy in ALBC. In the current study, we found varied differences in the elution capability of vancomycin when the types of bone cements were controlled. The possible explanation for this is that the byproducts of generic vancomycin aggregate in the ALBC, forming a different structure, pore size, and pore number within the PMMA. Another evidence suggests that an “impurity”, such as the like the byproducts of vancomycin, in the ALBC may influence antibiotic elution. When hand mixed, the release of vancomycin is greater in ALBC prepared using vancomycin and another antibiotic than using vancomycin alone. The effect is reduced when ALBC is mixed under vacuum condition [23, 34]. The phenomenon is explained by the fact that both antibiotics act as soluble additives that create cracks and voids in the ALBC, which increase the diffusion surface. When mixed in vacuum condition, the cracks and voids are minimized, so the effect is reduced.

During the setting of bone cement, pores are formed as a result of the chemical reaction and volume reduction, which are the starting points of cement breakage [5]. There are some significant differences in the mechanical strength of different brands of bone cement, but these differences are small compared with other factors, such as temperature, fat, or blood in the cement [10]. In general, the mechanical strength used for definite fixation of prosthesis must have ultimate compression strength of more than 70MPa as described by international industrial standard [9, 43]. In our study, the compression strength of high dose ALBCs after elution does not exceed 70MPa in Lyo-Vancin and Strerile Vancomycin groups, regardless of the brands of PMMA used. In contrast, the Vanco group exhibits enough mechanical strength before and after elution. In the low dose groups, which mimic the clinical scenario for ALBCs that are mainly for definite fixation, all combinations of different brands of PMMA and vancomycin show compression strength of more than 70MPa.

Some clinical studies compared the mechanical strength of high and low dose ALBC [8, 9, 44–46]. Some authors found that the addition of antibiotic to bone cement had no effect on the mechanical strength, while others reported a significant reduction. However, several factors would contribute to different results, such as brands of bone cement used, categories and amount of antibiotic, methods of antibiotic mixing and bone cement preparation, and methods of mechanical test [44]. Vancomycin in bone cement was studied less than gentamicin or tobramycin in the literature. Vancomycin is different in that it is an amphoteric substance with higher molecular weight. This could result in an interaction between the monomer and vancomycin, and subsequently lower molecular weight of final polymeric chains and weaker molecular strength [8]. Moreover, Topoleski et al. found that failure of ALBC usually begins with antibiotic agglomerations, which acts as stress risers [47]. Vancomycin, a much larger molecule than other antibiotics, might enhance the effect [8]. As a result, the findings in the current study that high dose ALBC has lower mechanical stress might be justified.

The literature reported contradictory results on the mechanical strength of bone cement after aging. Looney et al. reported that the strength increased in the first 1–2 weeks after the setting of bone cement [48]. Miola et al. found that the compression strength of ALBC after 14 days of elution was greater than that before elution [36]. The authors explained this phenomenon by the possible late-polymerization, post-hardening, and post-curing processes [9]. However, Ayre et al. reported reduced mechanical and fatigue properties of bone cement over time [49]. They proposed that with aging of bone cement, three processes occur simultaneously: cement has ongoing polymerization after setting and thus increases the strength; unreacted monomer and other substances diffuse out; water or body fluid penetrates in. In terms of bone cement loaded with antibiotics, the fourth process, antibiotics eluting from the antibiotic-bone cement construct, may decrease the mechanical strength. Accordingly, different bench-top settings would affect one or more of the three processes, thus changing the mechanical strength of bone cement after elution.

There are a number of limitations to this study. We did not perform a comprehensive test on the mechanical properties, such as fatigue strength and tensile strength. In addition, in the in vivo condition, continuous loading and unloading cycles may further increase the rate of degradation and weaken the mechanical strength. In addition, we used PBS as an elution solution in this *in vitro* study, which does not account for the in vivo conditions such as body fluid, antibiotic stability, and host response. Moreover, the mixing technique of bone cement is another issue, since PMMA mixed in vacuum condition would reduce porosity and stress risers. However, in the practical viewpoint, a proportion of surgeons continue using hand-mixing technique for ALBC preparation.

## Conclusions

Different brands of PMMA bone cements loaded with different brands of vancomycin can have different elution ability and mechanical strength. In terms of vancomycin release ability, PALACOS bone cement seems to have higher elution ability in high or low dose ALBC, while Lyo-Vancin and Sterile Vancomycin have higher elution ability in high and low dose ALBC, respectively. In terms of ultimate compressive strength, no single brand of bone cement exhibit superior results. In high dose groups, several combinations fail to achieve 70MPa as recommended for definite fixation after elution, while in low dose groups all combinations pass the threshold. Our study sheds light not only of the influence of different brands of bone cement, but also of the different brands of vancomycin on the ultimate compressive strength and antibiotic elution ability, which can be more than 5 fold. Future studies on the best combination of bone cements and antibiotics can be performed to achieve the highest infection control or prevention rate, while not compromising mechanical strength.

## Supporting Information

S1 Table Vancomycin elution in high dose group when brand of vancomycin is controlled. (XLS)

S2 Table Vancomycin elution in low dose group when brand of vancomycin is controlled. (XLSX)

S3 Table Vancomycin elution in high dose group when brand of bone cement is controlled. (XLSX)

S4 Table Vancomycin elution in low dose group when brand of bone cement is controlled. (XLSX)

S5 Table Compression strength. The tables show compression strength before and after elution in high and low dose group.
(XLSX)
